# Health Risk Behaviour among In-School Adolescents in the Philippines: Trends between 2003, 2007 and 2011, A Cross-Sectional Study

**DOI:** 10.3390/ijerph13010073

**Published:** 2015-12-23

**Authors:** Karl Peltzer, Supa Pengpid

**Affiliations:** 1 ASEAN Institute for Health Development, Mahidol University, Salaya, Phutthamonthon, Nakhon Pathom 73170, Thailand; supaprom@yahoo.com; 2 Department of Research & Innovation, University of Limpopo, Turfloop Campus, Sovenga 0727, South Africa; 3 HIV/AIDS/STIs and TB (HAST), Human Sciences Research Council, Pretoria 0001, South Africa

**Keywords:** monitoring, overweight, dietary intake, physical activity, sedentary behaviour, substance use, injury, violence, mental health, protective factors

## Abstract

Intermittent monitoring of health risk behaviours at the population level is important for the planning and evaluation of national health promotion intervention programmes. The study aimed to provide trend estimates on the prevalence of various health risk behaviours assessed in the Global School-based Health Survey in 2003, 2007 and 2011 in the Philippines. Three waves of cross-sectional data included 18,285 school-going adolescents, 47.4% male and 52.6% female, aged between 11 years or younger and 16 years or older, with a mean age of about 14.7 years (SD = 1.2), and mainly in second to fourth year study Grade. Significant improvements in health risk and risk behaviours (overweight or obese and smokeless tobacco use among boys, being in a physical fight, troubles from alcohol drinking, mental health, oral and hand hygiene among both boys and girls) but also increases in health risk behaviour (bullying victimization, injury and loneliness) among both boys and girls were found in this large study over a period of eight years in the Philippines. High prevalences of health risk behaviours and increases in some of them should call for intensified school health promotion programmes to reduce such risk behaviours.

## 1. Introduction

“In the Philippines, four out of the ten leading causes of deaths among youth and young adults aged 10–24 years are non-communicable in nature, and these are mostly attributable to risk behaviours” [1]. Monitoring trends in health risk behaviours can help in identifying potential preventive strategies [2]. Such health risk behaviours may include: (1) overweight and dietary behavior; (2) physical activity and sedentary behaviour; (3) substance use; (4) injury and violence; (5) poor mental health; (6) oral and hand hygiene and (7) protective factors.

A number of studies examined trends in health risk behaviour among adolescents (young people between the ages 10–19 years [3]) in high income countries, mostly over a 10 year period, utilizing mainly the Health Behaviour in School-aged Children (HBSC) surveys. Regarding (1) overweight and dietary behaviour, three studies (in Czech Republic, Germany and USA) found an increase of Body Mass Index (BMI) overweight from 2002–2010 or 2014 [4,5,6] and one study in USA an increase of the prevalence of fruit and vegetable consumption from 2002 to 2010 [6]. In terms of (2) physical activity and sedentary behaviour, three studies (in USA, Germany and in 32 countries in Europe and North America) [5,6,7] found an increase in physical activity of moderate-to-vigerous intensity (at least 60 min daily) from 2002 to 2010 and one study in Czech Republic [4] a decrease in physical activity of moderate-to-vigerous intensity (at least 60 min daily) from 2002 to 2014. In relation to (3) substance use, three studies in New Zealand, in 20 of 28 European and North American countries countries and in all geographic regions, and Germany [2,8,9] found a decrease in substance use (alcohol, tobacco) from 2002 to 2010, or 2001 to 2012. For (4) injury and violence, a reduction in bullying victimization was observed in New Zealand from 2001 to 2012 [2], and in a third of 33 countries in Europe and North America from 2002 to 2010 [10,11], and a reduction in fighting in New Zealand from 2001 to 2012 [2]. Regarding (5) poor mental health, in Switzerland, relative to 1994, lower levels of psychological health complaints (“feeling low”, “irritability and bad temper”, “nervousness” and “difficulties in getting to sleep”) were experienced in 1998, 2002 and 2006 [12], while in New Zealand depressive symptoms did not improve from 2001 to 2012.” [2] In terms of (6) oral and hand hygiene, the prevalence of poor toothbrushing frequency (<twice/day) in 20 countries decreased between 1994 and 2010 in most of the countries and regions from 38%–70% to 28%–50% [13]. In relation to (7) protective factors, positive connections to school and family improved in New Zealand [2].

Few national studies among adolescents examining health risk behaviour trends in low and middle income countries have been conducted. Regarding overweight and physical activity, overweight and obesity prevalence among school going 13–15 year olds in Argentina significantly increased from 24.5% and 4.4% in 2007 to 28.6% and 5.9% in 2012, respectively, and the level of physical activity increased from 12.7% in 2007 to 16.7% in 2012 [14]. In terms of substance use, the prevalence of tobacco use among adolescents reduced from 24.5% in 2007 to 19.6% in 2012 in Argentina [14]. Likeweise, data from the Global Youth Tobacco Survey (GYTS) in 13–15-year-old students in the Philippines show a decrease of current tobacco use from 27.1% in 2000 to 13.7% in 2011 [15,16]. Further, based on data from the GYTS in the South-East Asia region, the prevalence of current smokeless tobacco use increased in Bhutan from 9.4% in 2009 to 23.2% in 2013, and in Nepal from 6.1% in 2007 to 16.2% in 2011 [17]. Regarding injury and violence, self-reported serious injury among adolescents in Morocco decreased from 44.7% in 2006 to 29.5% in 2010 [18], and bullying victimization increased among adolescents in Venezuela from 33.4% in 2004 to 43.6% in 2008 [19]. As a protective factor, school attendance (no truancy) decreased in school-going adolescents in Venezuela from 89.2% in 2004 to 79.2% in 2008 [19]. 

The study aimed to provide trend estimates on the prevalence of various health risk behaviours assessed in the Global School-based Health Survey (GSHS) in 2003, 2007 and 2011 in the Philippines. It is hypophesized that overweight and dietary behaviour, physical activity and sedentary behaviour, substance use, injury and violence, poor mental health, oral and hand hygiene and protective factors may have changed from 2003 to 2011. The study findings could give insights on trends in the prevalence of health behaviours and protective factors for use in the evaluation of school health and youth health promotion prevention strategies [1].

## 2. Methodology

### 2.1. Description of Survey and Study Population

This study was a secondary analysis of existing data from the GSHS from Philippines. GSHS details and data can be accessed in [19]. The 2003, 2007 and 2011 Philippines GSHS used a two-stage (schools and classrooms) cluster sampling design to generate a nationally representative sample of students in year 1 to 4 in secondary schools [1]. Students completed a self-administered questionnaire under the supervision of trained survey administrators [1]. “A weighting factor was applied to each student record to adjust for non-response and for the varying probabilities of selection. The weighting formula used for calculation was: W = W1 × W2 × f1 × f2 × f3; W1 = inversion of probability of selecting each school, W2 = inversion of probability of selecting each class room, f1 = adjustment factor for non-response at school level, f2 = adjustment factor for non-response at class level, f3 = A post stratification adjustment factor calculated by sex within grade.” [1]. The frequency of missing values was between 0.6%–19.6% in the multivariate models. Cases with missing values were excluded from the analysis.

### 2.2. Measures

The study variables used were from the GSHS [20] are described in Table A1. Body weight and height were recorded by self-report, and obesity was classified as children with BMI figures referring to an adult BMI of ≥30.0 kg/m^2^ using international age- and gender-specific criteria [21]. The GSHS questionnaire was found to have good validity in a previous validation study [22]. Inadequate fruit consumptions was defined as less than two or more servings a day and inadequate vegetable consumption as less than three or more servings a day [23]. The two physical activity questions and have been tested for validity and reliability [24]. Cronbach alpha for this two item physical activity measure was 0.83 in the 2003 GSHS and 0.76 in the 2007 GSHS in the Philippines; in the 2011 GSHS in the Philippines physical activity was only assessed with one question (the first item). Inadequate physical activity was defined as obtaining less than 60 min of physical activity per day on at least 5 days per week [24,25]. “Sedentary” behaviour was defined as spending 3 or more hours per day sitting [25].

### 2.3. Data Analysis

Data analysis was conducted using STATA software version 12.0 (Stata Corporation, College Station, TX, USA). This software provides robust standard errors that account for the sampling design, *i.e.*, cluster sampling owing to the sampling of school classes. Logistic regression analyses were conducted for boys and girls separately with each outcome regressed on year of study, age, overweight and dietary behaviour, physical activity and sedentary behaviour, substance use, injury and violence, poor mental health, oral and hand hygiene and protective factors. When significant effects (*p* < 0.05) were detected, interactions of study year with age were added to the model. In reporting, weighted percentages are given, and the sample size reported reflects the sample that was asked the target question. The two-sided 95% confidence intervals are reported, and *p*-values less or equal to 5% are used to indicate statistical significance. The reported 95% confidence intervals and the *p*-values are both adjusted for the multistage stratified cluster sample design of the survey.

## 3. Results 

### 3.1. Sample Characteristics

The three waves of the Philippine sample of the GSHS included 18285 school-going adolescents, 47.4% male and 52.6% female, aged between 11 years or younger and 16 years or older, with a mean age of about 14.7 years (SD = 1.2), and mainly in second to fourth year study Grade. The survey response rates were 84% in 2003, 81% in 2007 and 82% in 2011 [19]. Sample sizes and weighted demographic characteristics for all three cohorts are shown in Table 1. Across the study samples, there was an increasing proportion of male students and decreasing number of female students (*p* < 0.001). The 2007 sample was older than the samples in 2003 and 2011 (*p* < 0.001). All subsequent analyses controlled for sample demographic characteristics (see Table 1).

### 3.2. Overweight and Dietary Intake

The proportion of male students classified as overweight or obese decreased significantly from 16.6% in 2003 to 9.7% in 2011, while this did not change among girls. A large proportion ate less than two servings of fruits a day (61.8% in boys and 58.9% in girls) and less than three servings of vegetables per day (74.4% in boys and 77.7% in girls) in 2003, which did not change in 2007 and 2011. The number of students who went mostly or always hungry decreased from 9.6% in boys and 6.4% in girls in 2003 to 8.1% in boys and 5.4% in girls, respectively in 2011, but this was not significant (see Table 2 and Table 3).

**Table 1:** Characteristics of participating students for 2003, 2007 and 2011 surveys (*N* = 18,285).

Variable	2003 (*N* = 7338)	2007 (*N* = 5657)	2011 (*N* = 5290)
	*N* (%)	*N* (%)	*N* (%)
Gender			
Male	3094 (43.2)	2449 (47.2)	2279 (49.5)
Female	4188 (56.8)	3190 (52.8)	2986 (50.5)
Missing	56	18	25
Age in years			
11 years or younger	21 (0.3)	17 (0.3)	20 (0.5)
12	38 (1.0)	51 (0.7)	205 (5.7)
13	784 (15.9)	469 (7.8)	980 (20.0)
14	1518 (27.2)	1293 (22.4)	1350 (22.0)
15	1858 (26.8)	1671 (30.4)	1310 (24.8)
16 years or older	2895 (28.8)	2097 (38.4)	1384 (27.0)
Missing	224	59	91
Grade			
First year	29 (0.6)	262 (3.2)	1246 (28.9)
Second year	2228 (40.4)	1991 (33.2)	1832 (26.2)
Third year	1978 (32.0)	1704 (33.6)	1112 (23.8)
Fourth year	3021 (27.0)	1608 (30.0)	1037 (21.1)
Missing	82	92	63

**Table 2:** Health risk behaviours in 2003, 2007 and 2011 among male in-school adolescents in the Philippines.

Variable	2003	2007	2011	Change over Time Compared to 2003	
	N (%)	N (%)	N (%)	2007 Adjusted ^1^ OR (95% CI)	2011 Adjusted ^1^ OR (95% CI)
***Overweight and dietary behaviour***					
Overweight or obesity	310 (16.6)	240 (10.7)	187 (9.7)	0.70 (0.40–1.23)	0.56 (0.34–0.92) *
Fruits <2 day	1912 (61.8)	1672 (63.8)	1414 (62.2)	0.73 (0.33–1.63) **^2^**	1.15 (0.60–2.19)
Vegetable <3 day	2379 (74.4)	1931 (78.1)	1706 (73.5)	1.23 (0.97–1.57)	1.04 (0.81–1.35)
Went hungry (mostly/always)	367 (9.6)	239 (8.3)	195 (8.1)	0.99 (0.70–1.41)	0.99 (0.69–1.41)
***Physical activity and sedentary behaviour***					
Physical activity (≤5 days/week of 60 min)	2599 (87.3)	2203 (91.0)	1906 (86.4)	1.56 (1.15–2.12) **	1.03 (0.74–1.45)
Time sitting 3 or more hours/day	736 (26.5)	647 (27.7)	595 (29.8)	1.04 (0.73–1.50)	1.21 (0.82–1.77)
***Substance use***					
Smoking cigarettes	688 (23.5)	475 (22.9)	445 (20.4)	0.83 (0.61–1.13)	0.85 (0.62–1.17)
Other tobacco products	418 (10.7)	170 (6.8)	154 (6.2)	0.60 (0.40–0.90) *	0.65 (0.42–0.99) *
Tobacco use (self, past month)	750 (25.6)	508 (24.6)	485 (22.1)	0.55 (0.16–1.96) **^2^**	0.80 (0.30–2.12)
Tobacco use (any parent/guardian)	1297 (43.0)	1018 (42.9)	842 (38.5)	1.00 (0.84–1.21)	0.89 (0.73–1.09)
Alcohol use (in past month)	973 (31.6)	621 (30.2)	597 (28.7)	0.81 (0.62–1.05)	0.93 (0.73–1.18)
Drunk in life time	1115 (32.5)	762 (33.0)	525 (24.8)	0.88 (0.70–1.10)	0.71 (0.56–0.90) **
Trouble from drinking	724 (22.2)	567 (25.2)	213 (10.0)	1.01 (0.35–2.91) **^2^**	0.34 (0.13–0.90) *
***Injury and violence***					
Any serious injury	699 (39.1)	1083 (54.2)	1030 (54.3)	2.22 (1.72–2.86) ***	2.28 (1.83–2.83) ***
Bullied	960 (34.7)	887 (44.3)	964 (46.0)	1.69 (1.32–2.17) ***	1.72 (1.35–2.20) ***
In physical fight	1594 (51.6)	984 (41.5)	897 (42.8)	0.67 (0.53–0.84) ***	0.72 (0.57–0.90) **
***Poor mental health***					
Had no close friends	121 (4.0)	117 (4.7)	86 (4.4)	1.42 (0.76–2.67)	1.44 (0.84–2.47)
Seriously thinking about suicide (past 12 months)	611 (18.4)	335 (14.1)	264 (12.0)	0.80 (0.56–1.04)	0.57 (0.40–0.81) **
Suicide plan (past 12 months)	601 (16.6)	183 (7.5)	216 (9.6)	0.56 (0.39–0.81) **	0.68 (0.48–0.96) *
Lonely (mostly/always)	345 (9.5)	333 (14.4)	290 (12.7)	1.75 (1.12–2.73) *	1.51 (1.00–2.30) *
Could not sleep (mostly/always)	408 (12.8)	279 (12.1)	239 (10.4)	1.17 (0.86–1.60)	0.90 (0.67–1.21)
***Oral and hand hygiene***					
Brushed teeth (<twice/day)	1339 (42.4)	402 (14.4)	303 (12.2)	0.18 (0.12–0.27) ***	014 (0.10–0.21) ***
Wash hands before eating (not always)	1196 (40.5)	916 (37.5)	835 (37.3)	1.05 (0.55–2.03)	1.03 (0.58–1.83) **^2^**
Wash hands after toilet/latrine use (not always)	1046 (29.7)	685 (27.5)	563 (23.1)	0.92 (0.74–1.14)	0.77 (0.60–0.99) *
Wash hands with soap (not always)	1584 (50.5)	1129 (45.3)	1039 (43.9)	0.42 (0.22–0.79) ^2^^,^**	0.55 (0.34–0.90) *
***Protective factors***					
Truancy	1144 (35.6)	895 (39.4)	806 (37.1)	1.08 (0.74–1.56)	1.35 (0.93–1.96)
Peer support (mostly/always)	840 (28.8)	672 (28.2)	687 (28.5)	0.79 (0.60–1.05)	0.83 (0.61–1.13)
Parents/guardians supervision (mostly/always)	634 (21.7)	485 (20.6)	473 (20.6)	1.03 (0.74–1.44)	0.83 (0.63–1.08)
Parents/guardians connectedness (mostly/always)	752 (25.4)	586 (23.6)	555 (25.3)	0.99 (0.81–1.21) **^2^**	0.99 (0.81–1.19)
Parents or guardians bonding (mostly/always)	800 (28.7)	588 (25.2)	603 (26.5)	0.90 (0.72–1.13) **^2^**	0.88 (0.69–1.11)

* *p* < 0.05; ** *p* < 0.01; *** *p* < 0.001; **^1^** Adjusted for age, overweight and dietary behaviour, physical activity and sedentary behaviour, substance use, injury and violence, poor mental health, oral and hand hygiene and protective factors;**^2^** Interaction Time × Age *p* < 0.05.

**Table 3:** Health risk behaviours in 2003, 2007 and 2011 among female in-school adolescents in the Philippines.

Variable	2003	2007	2011	Change over Time Compared to 2003	
	N (%)	N (%)	N (%)	2007 Adjusted ^1^ OR (95% CI)	2011 Adjusted ^1^ OR (95% CI)
***Overweight and dietary behaviour***					
Overweight or obesity	293 (11.4)	170 (6.8)	247 (9.4)	0.63 (0.33–1.21)	0.79 (0.47–1.33)
Fruits <2 day	2506 (58.9)	1985 (61.5)	1859 (61.2)	1.16 (0.95–1.41)	1.13 (0.95–1.34)
Vegetable <3 day	3255 (77.7)	2508 (78.7)	2266 (74.7)	1.10 (0.90–1.35)	0.87 (0.68–1.10)
Went hungry (mostly/always)	305 (6.4)	236 (6.6)	176 (5.4)	1.10 (0.74–1.63)	0.91 (0.61–1.35)
***Physical activity and sedentary behaviour***					
Physical activity (≤5 days/week of 60 min)	3665 (89.6)	2946 (92.8)	2531 (86.7)	1.84 (1.37–2.46) ***	0.89 (0.64–1.24)
Time sitting 3 or more hours/day	1150 (31.6)	976 (32.7)	900 (33.9)	1.02 (0.74–1.39)	1.07 (0.77–1.50)
***Substance use***					
Smoking cigarettes	255 (8.2)	191 (7.8)	164 (7.7)	0.99 (0.63–1.54)	1.00 (0.64–1.55)
Other tobacco products	115 (2.5)	48 (1.7)	43 (1.6)	0.37 (0.15–0.94) *	0.95 (0.42–1.98)
Tobacco use (self, past month)	283 (8.9)	205 (8.4)	181 (8.2)	0.83 (0.49–1.40)	0.94 (0.55–1.62)
Tobacco use (any parent/guardian)	1903 (44.1)	1462 (46.0)	1229 (43.4)	1.10 (0.91–1.33)	1.06 (0.87–1.30)
Alcohol use (in past month)	752 (17.9)	412 (16.1)	427 (17.9)	0.70 (0.47–1.04)	0.85 (0.64–1.12)
Drunk in life time	806 (18.0)	520 (18.2)	401 (16.6)	0.76 (0.55–1.06)	0.88 (0.60–1.06)
Trouble from drinking	572 (14.3)	443 (14.6)	164 (7.1)	0.88 (0.57–1.07)	0.42 (0.29–0.59) ***
***Injury and violence***					
Any serious injury	711 (25.9)	1169 (42.2)	1062 (42.3)	2.22 (1.79–2.76) ***	2.27 (1.77–2.90) ***
Bullied	1344 (36.1)	1237 (47.0)	1420 (49.1)	1.68 (1.35–2.09) ***	1.79 (1.45–2.20) ***
In physical fight	2004 (48.8)	1083 (32.9)	866 (30.6)	0.54 (0.44–0.66) ***	0.46 (0.38–0.57) ***
***Poor mental health***					
Had no close friends	113 (2.5)	125 (4.2)	79 (2.8)	1.74 (0.96–3.18)	1.60 (0.86–2.96)
Seriously thinking about suicide (past 12 months)	752 (16.2)	663 (20.9)	593 (21.8)	1.35 (1.00–1.82) *	1.57 (1.16–2.14) **
Suicide plan (past 12 months)	760 (16.8)	314 (10.2)	374 (13.8)	0.55 (0.40–0.77) ***	0.76 (0.56–1.05)
Lonely (mostly/always)	477 (11.2)	664 (22.2)	491 (16.9)	3.19 (2.25–4.52) ***	2.32 (1.15–3.25) ***
Could not sleep (mostly/always)	579 (16.1)	435 (15.1)	345 (12.4)	0.88 (0.71–1.10)	0.69 (0.54–0.88) **
***Oral and hand hygiene***					
Brushed teeth (<twice/day)	1466 (32.4)	301 (7.4)	250 (8.5)	0.14 (0.10–0.21) ***	0.14 (0.10–0.20) ***
Wash hands before eating (not always)	1377 (35.0)	1141 (34.6)	1094 (37.4)	0.93 (0.72–1.22)	1.05 (0.82–1.34)
Wash hands after toilet/ atrine use (not always)	1267 (27.2)	853 (24.1)	707 (22.3)	0.76 (0.63–0.94) *	0.76 (0.61–0.96) *
Wash hands with soap (not always)	1803 (42.9)	1285 (37.9)	1257 (43.1)	0.81 (0.67–0.97) *	0.95 (0.78–1.16)
***Protective factors***					
Truancy	1208 (28.0)	1008 (33.7)	837 (30.7)	1.52 (1.01–2.27) *	1.46 (0.95–2.29)
Peer support (mostly/always)	1217 (31.3)	966 (31.4)	1015 (31.9)	0.97 (0.71–1.32)	0.93 (0.68–1.27)
Parents or guardians supervision (mostly/always)	980 (24.0)	687 (21.4)	669 (22.2)	0.84 (0.64–1.09)	0.78 (0.61–1.01)
Parents/guardians connectedness (mostly/always)	1207 (29.7)	841 (26.6)	860 (28.9)	0.71 (0.48–1.06)	1.07 (0.69–1.65) **^2^**
Parents or guardians bonding (mostly/always)	1430 (37.8)	1094 (34.7)	1053 (35.1)	0.83 (0.66–1.05)	0.83 (0.66–1.04)

* *p* < 0.05; ** *p* < 0.01; *** *p* < 0.001; **^1^** Adjusted for age, overweight and dietary behaviour, physical activity and sedentary behaviour, substance use, injury and violence, poor mental health, oral and hand hygiene and protective factors; **^2^**Interaction Time x Age *p* < 0.05.

### 3.3. Physical Activity and Sedentary Behaviour

A large majority (86%–93%) among both boys and girls had less than five days a week, 60 min a day of moderate-to-vigerous physical activity, which increased from 2003 to 2007 but returned to 2003 levels in 2011. The time of sitting three or more hours per day increased but not significantly among boys from 26.5% in 2003 to 29.8% in 2011 and among girls from 31.6% in 2003 to 33.9% in 2011.

### 3.4. Substance Use

A significant proportion of male students currently smoked cigarettes (23.5%, 8.2% in girls) and currently used any tobacco products (25.6%, 8.9% in girls), which did not change over time. However, the use of other tobacco products such as chewing tobacco leaves significantly decreased among boys (not girls) from 2003 (10.7%) to 2011 (6.2%). The prevalence of over 40% tobacco use among parents or guardians of the stdents did not change over time. Lifetime drunkenness decreased significantly among boys (not girls) from 32.5% in 2003 to 24.8% in the assessment year of 2011. There was a sharp decrease in ever having had a hangover, felt sick, got into trouble with their family or friends, missed school, or got into fights, as a result of drinking alcohol among boys and girls, repectively, from 22.2% and 14.3% in 2003 to 10.0% and 7.1% in 2011.

### 3.5. Injury and Violence

The annual unintentional injury prevalence seems to have increased significantly in both boys and girls from 2003 (39.1% in boys and 25.9% in girls) to 2011 (54.3% in boys and 42.3% in girls). Having been bullied also increased significantly in both boys and girls from 2003 to 2011. On the other hand, having been in a physical fight decreased in both boys and girls from 2003 to 2011.

### 3.6. Poor Mental Health

Compared to 2003, suicidal ideation (making a suicide plan and seriously thinking about it) among boys significantly reduced in 2007 and 2011, while seriously thinking about suicide increased among girls from 2003 (16.2%) to 2011 (21.8%). There was a steady significant increase of being lonely over the three assessment points. Anxiety or worry symptoms significantly decreased among girls but not boys in 2011 compared to 2003 and 2007. No significant differences were observed regarding having no close friends.

### 3.7. Oral and Hand Hygiene

Compared to 2003 (42% in boys and 32.4% in girls) poor tooth brushing (<twice/day) declined significantly in 2007 (14.4% in boys and 7.4% in girls) and 2011 (12.2% in boys and 8.5% in girls). Poor hand washing after toilet or latrine use (not always) also reduced significantly among both boys and girls over time. However, poor hand washing before eating (not always) and not always washing hands with soap remained high among boys (although it reduced) and girls at around 40% over time.

### 3.8. Protective Factors

School attendance, peer support and parental or guardian support were similar among both boys and girls across all three assessment points (see Table 2 and Table 3).

## 4. Discussion

The study found in three nationally representative adolescent school going surveys in 2004, 2007 and 2011 in the Philippines decreases in being overweight or obese (among boys), use of smokeless tobacco (among boys), being in a physical fight, troubles from alcohol drinking, poor mental health (suicidal ideation to make a suicide plan among boys and worry or anxiety among girls), oral and hand hygiene, while bullying victimization, having sustained a serious injury and being lonely increased over the three assessment periods. Some of the improvements may be associated with school-based health promotion and other public health strategies [26,27]. Unlike a number of previous studies [4,5,6,14] that found an increase in adolescent overweight or obesity over time, this study found decreases in being overweight or obese among boys in the Philippines. Contrary to several previous studies [2,14,28], smoking did not decline over time in the Philippines but the use of smokeless tobacco among boys did, again unlike in Bhutan and Nepal where smokeless tobacco use increased [17]. Comparing the prevalences of this study (GSHS) with the study findings from the GYTS, which was conducted in similar study years 2004, 2007 and 2011 as the GSHS, the GYTS found declines in smoking in 2011 (8.9% compared to 14.6% in 2011 in the GSHS) and no decline in smokeless tobacco use in 2011 (7.3% compared to 3.9% in 2011 in the GSHS) [29,30].

Adolescent alcohol use did not decline, as found in European and North American countries [8], but troubles from alcohol drinking declined. This could mean that students may have learnt to drink alcohol more in moderation so as to avoid troubles from drinking. In this context, reported reductions in fighting over time, as also reported in some other studies [2], may be related to drinking related fighting reductions. In agreement with a study in Switzerland [12] lower levels of psychological distress (suicide plan, anxiety) were found over time, while loneliness significantly increased.

Physical activity level meeting recommendations were low (below 13.5%) and did not change over time. This is lower than in across 32 countries from Europe and North America [7] and one of the lowest in 34 GSHS survey countries [25] and calls for interventions targeting the increase of physical activity. Although in a number of HBSC survey countries bullying victimization had declined [10,11], there was a stark increase from 35.7% in 2003 to 47.7% in 2011 in the Philippines. A similar increase in bullying victimization (33.4% in 2004 and 43.6% in 2008) was found in the Venezuela GSHS [19]. The bullying victimization prevalence in the Philippines seems much higher than the global rate of 30% among adolescents and together with Indonesia the highest in the region [31]. The Philippines government should invest more systematically in the prevention effort on bullying. Likewise, there was an even greater increase in the annual prevalence of having sustained a serious injury from 2003 to 2011. On the other hand, in Morocco, self-reported serious injury among school adolescents decreased from 2006 to 2010 [18]. In the study region similar annual prevalences of serious injury were found in Indonesia and Thailand [32], calling for increased injury prevention and safety promotion activities among school children in the region [33]. 

Oral hygiene (tooth brushing) improved very significantly among adolescents over time in the Philippines. Similar improvements have been found in tooth brushing behaviour over time in European countries [13]. In terms of hand hygiene behaviour improvements in handwashing after toilet or latrine use, but not other hand hygiene behaviour (before eating) were found, similar to rates found in countries of the Southeast Asian region [34]. These dramatic improvements in oral hygiene behaviour and some partial improvements in hand hygiene behaviour may be attributed to an essential health care package for children (the “Fit for School” program in the Philippines) [35]. Inadequate fruit and vegetable consumption was high, as also found in other countries in the region [36] and did not improve over time, contrary to what was found, for example among U.S. students [6]. Protective factors (school attendance, peer and parental support) did not change over time, while in New Zealand positive connections to school and family improved [2].

### Limitations of the Study

This study had several limitations. Firstly, the GSHS only enrols adolescents who are in school. The secondary school enrollment ratio was 69% in 2013 in the Philippines [37]. School-going adolescents may not be representative of all adolescents in a country as the occurrence of health risk behaviours may differ between the two groups. The data were based on self-report, which may have introduced bias. Finally, the cross-sectional data do not provide information about causal relationships.

## 5. Conclusions

Significant improvements in health risk and risk behaviours (overweight or obese and smokeless tobacco use among boys, being in a physical fight, troubles from alcohol drinking, mental health, oral and hand hygiene among both boys and girls) but also increases in health risk behaviour (bullying victimization, injury and loneliness among both boys and girls) were found in this large study over a period of eight years in the Philippines. High prevalences of health risk behaviours and increases in some of them should call for intensified school health promotion programmes to reduce such risk behaviours.

## Appendix A

**Table A1:** Variable description.

Variables	Question	Response Options
*Physical activity*	“During the past 7 days, on how many days were you physically active for a total of at least 60 min per day?”	0 = 0 days to 8 = 7 days
	“During a typical or usual week, on how many days are you physically active for a total of at least 60 min per day?”	0 = 0 days to 8 = 7 days
*Sitting (excluding when in school or doing homework)*	“How much time do you spend during a typical or usual day sitting and watching television, playing computer games, talking with friends, or playing cards?”	1 = Less than 1 h per day… 3 = 3 to 4 hours per day …6 = 8 or more hours a day
*Hunger*	“During the past 30 days, how often did you go hungry because there was not enough food in your home?”	1 = never to 5 = always
*Fruits*	“During the past 30 days, how many times per day did you usually eat fruit, such as bananas, mangos and papayas?”	1 = I did not eat fruit during the past 30 days to 7 = 5 or more times per day
*Vegetables*	“During the past 30 days, how many times per day did you usually eat vegetables, such as tomatoes, kangkong, cabbage and string beans?”	I did not eat vegetables during the past 30 days to 7 = 5 or more times per day
*Height*	“How tall are you without your shoes on?”	
*Weight*	“How much do you weigh without your shoes on?”	
*Current smoking cigarettes*	“During the past 30 days, on how many days did you smoke cigarettes?”	1 = 0 days to 7 = All 30 days
*Current other tobacco use*	“During the past 30 days, on how many days did you use any other form of tobacco, such as chewing tobacco leaves?”	1 = 0 days to 7 = all 30 days
*Parental tobacco use*	“Which of your parents or guardians use any form of tobacco?”	1 *=* Neither; 2 = My father or male guardian; 3 = My mother or female guardian; 4 = Both parents
*Current alcohol use*	“During the past 30 days, on how many days did you have at least one drink containing alcohol?”	1 = 0 days to 7 = All 30 days
*Drunk*	“During your life, how many times did you drink so much alcohol that you were really drunk?“	1 = 0 times to 4 = 10 or more times
*Trouble from drinking*	“During your life, how many times have you ever had a hangover, felt sick, got into trouble with your family or friends, missed school, or got into fights, as a result of drinking alcohol?”	1 = 0 times to 4 = 10 or more times
*Bullied*	“During the past 30 days, on how many days were you bullied?”	1 = 0 days to 7 = All 30 days
*In physical fight*	“During the past 12 months, how many times were you in a physical fight?”	1 = 0 times to 8 = 12 or more times
*Injury*	“During the past 12 months, how many times were you seriously injured?” (An injury is serious when it makes you miss at least one full day of usual activities (such as school, sports, or a job) or requires treatment by a doctor or medical personnel.)“	1 = 0 times 8 = 12 or more times
Mental health		
*Close friends*	“How many close friends do you have?”	1 = 0 to 4 = 3 or more
*Suicidal ideation*	“During the past 12 months, did you ever seriously consider attempting suicide?”	1 = yes, 2 = no
	“During the past 12 months, did you make a plan about how you would attempt suicide?”	1 = yes, 2 = no
*Lonely*	“During the past 12 months, how often have you felt lonely?”	1 = never to 5 = always
*Worry/anxiety*	“During the past 12 months, how often have you been so worried about something that you could not sleep at night?”	1 = never to 5 = always
Oral and hand hygiene		
*Oral hygiene*	“During the past 30 days, how many times per day did you usually clean or brush your teeth?”	1 = zero to 4 or more times per day
*Hand hygiene*	“During the past 30 days, how often did you wash your hands before eating?”	1 = never to 5 = always
	“During the past 30 days, how often did you wash your hands after using the toilet or latrine?”	1 = never to 5 = always
	“During the past 30 days, how often did you use soap when washing your hands?”	1 = never to 5 = always
Protective factors		
*Truancy*	“During the past 30 days, on how many days did you miss classes or school without permission?”	1 = 0 days to 10 or more days
*Peer support*	“During the past 30 days, how often were most of the students in your school kind and helpful?”	1 = never to 5 = always
*Parental or guardian supervision*	“During the past 30 days, how often did your parents or guardians check to see if your homework was done?”	1 = never to 5 = always
*Parental or guardian connectedness*	“During the past 30 days, how often did your parents or guardians understand your problems and worries?”	1 = never to 5 = always
*Parental or guardian bonding*	“During the past 30 days, how often did your parents or guardians really know what you were doing with your free time?”	1 = never to 5 = always
